# Flood susceptibility mapping utilizing the integration of geospatial and multivariate statistical analysis, Erbil area in Northern Iraq as a case study

**DOI:** 10.1038/s41598-023-39290-4

**Published:** 2023-07-24

**Authors:** Alaa Ahmed, Ali Al Maliki, Bassim Hashim, Dalal Alshamsi, Hasan Arman, Ahmed Gad

**Affiliations:** 1 Geosciences Department, United Arab Emirates University, 15551 Al Ain, United Arab Emirates; 2 National Water and Energy Center, United Arab Emirates University, 15551 Al Ain, United Arab Emirates; 3 Geology Department, Division of Water Resource, Desert Research Center, Mathaf El Matariya Street, Cairo, 11753 Egypt; 4 Ministry of Science and Technology, Environment, Water and Renewable Energy Directorate, Baghdad, 765 Iraq; 5 Geology Department, Faculty of Science, Ain Shams University, Cairo, 11566 Egypt

**Keywords:** Environmental sciences, Natural hazards

## Abstract

Climate extreme events such as floods and droughts in any area have a significant impact on human life, infrastructure, agriculture, and the economy. In the last two years, flash floods caused by heavy rainstorms have become frequent and destructive in many catchments in Northern Iraq. The present study aims to examine flash floods in the Erbil region, Northern Iraq using Remote sensing (RS), Geographic Information System (GIS), and Principal Component Analysis (PCA) for geomorphic data. PCA results revealed that 12 geomorphic parameters exhibited a significant correlation with two different statistical components. To facilitate practical application, ranks are assigned based on the calculated parameters for flood susceptibility mapping. Out of the 24 basins in the current study, three basins (16, 3, and 14) have the highest geomorphometric values (36–39), indicating the zone most susceptible to flash floods and making up a maximum area of 38.58% of the studied region. Six basins (4, 8, 9, 10, 12, and 15), which have geomorphometric values between 30 and 35 and cover a land area of 27.86%, are the most moderately vulnerable to floods. The remaining basins, which make up 33.47% of the research, are occasionally subject to floods and have geomorphometric scores below 30. The precision of the flood susceptibility mapping was validated using the bifurcation ratio and drainage density relationship as well as past flood damages, such as economic losses and human casualties. Most of the recorded injuries and fatalities took place in areas that were particularly prone to severe past flooding. Additionally, the investigation revealed that 44.56% of all populated areas are located in extremely vulnerable basins. The findings demonstrate a notable correlation between the identified flood-susceptible areas and the occurrence of past flood damage.

## Introduction

In recent decades, the impact of climate change has become increasingly observable. Hydro-meteorological disasters, for example, have become increasingly frequent and destructive worldwide^1^. One-third of all the global geophysical disasters in the world are floods, which have a significant negative impact on infrastructure, economy, and population^2–4^. Location, population, socioeconomic and cultural circumstances, institutional and political measures, as well as coping mechanisms that distinguish the impacts on individuals and human systems, have all been demonstrated to affect how vulnerable societies are to the effects of these disasters^5^, other factors that affect the level and scope of damage caused by a disaster in a place, including its susceptibility profile and features (frequency, duration, depth, and spatial extent)^6^. In this situation, vulnerability is predicated on the interactions of socioeconomic and environmental physical elements.

The Middle East and Northern Africa (MENA) region is among the most vulnerable to the potential impacts of climate change^7^. It is expected that the region will suffer from higher temperatures and intense heat waves affecting inhabitants and crop yields and will also affect marine ecosystems and fisheries^8^. In the MENA region, less but more intense rainfall, coupled with higher temperatures will likely cause more droughts and greater flooding, sea level rise, more intense cyclones, and new areas exposed to dengue, malaria, and other vector and waterborne diseases. Therefore, determining the risks, vulnerabilities, and hazards associated with flooding has become critical. The increase in flooding incidents can be primarily attributed not only to climatological factors associated with heavy rainfall events, but also to the geomorphic features, land use changes, population expansion, and urbanization^9,10^. Moreover, geological and structural features considerably influence the predictability of floods^11–13^. The analytical hierarchy process, fuzzy logic, and genetic algorithms, as well as decision tree models, multivariate statistics, and the hydrological forecasting system, have all been developed as tools for analyzing flood risk. Thus, analyzing geomorphic parameters could aid in predicting floods, sediment yields, and erosion rates^14–17^. The morphometric parameters have been introduced for measuring and mathematically analyzing the characteristics associated with the drainage basin^18^. Recently, the assessment of basin morphometry has improved in terms of accuracy, speed, and cost owing to the introduction of geographic information systems (GISs), remote sensing (RS), and high-resolution digital elevation models (DEMs)^19–21^. Several studies have demonstrated the potential of integrating RS and GIS for geomorphometric analysis of drainage basins^14,22,23^. This method is being used to develop useful, precise, and affordable techniques for defining flood susceptibility zones^14,24–26^. This method aids in determining the overall characteristics of the drainage basins, including their terrain and elevation, as well as the relationship between morphological factors and flood susceptibility within the drainage basin. When determining this interrelationship, it is crucial to use multivariate statistical analysis, such as principal component analysis (PCA), to reduce dimensionality in large data sets. Multivariate methods are commonly used in hydrological research to provide solutions and options for a specific problem^27^. It has been used to develop hydrological prediction equations involving geomorphic parameters, identifying the interrelationship between the different parameters and their effect on flood susceptibility^28–31^. The novelty of this study is a multi-disciplinary evaluation approach that combines geospatial data, statistical analysis and knowledge of flood hazards to improve the future adaptation plans. Similar physical and socio-economic characteristics, as well as flood-prone areas worldwide, could lead to the replication of such a hydro-social analytic approach.

Iraq is classified as one of the most environmentally vulnerable and fragile countries in the world^32^. The country is placed at the heart of the violent effects of climate extremes. Flash floods induced by heavy rainfall events and urbanization have become common and destructive, especially in the northern areas of the country.

Recently, the failure to respond to devastating flash floods in Erbil, Northern Iraq, was mainly attributed to the lack of available information about the flood-vulnerable areas. This caused huge economic damage and public outrage in the region. Following the coalition invasion of the country in 2003, northern areas serve as home to a significant population and host several resources that significantly contribute to the economy^33^. Consequently, dramatic urbanization, landcover change, and a significant increase in surface runoff become evident. The runoff in built-up areas increased from approximately 4 million cubic meters in 1990 to approximately 14 million cubic meters in 2016 and is expected to have increased by 2020^34^. Therefore, identifying and assessing flood susceptibility can effectively help raise awareness among the people living in flood-prone areas to reduce damage, fatalities, and losses. The objectives of the present study are as follows: (1) identify and evaluate geomorphological parameters; (2) investigate the multicollinearity of relationships between variables to eliminate less controlling factors from the analysis; (3) combine the remaining data using PCA for easier interpretation; and (4) estimate and evaluate flash flood risk in the northern Iraq region.

## Methods

### Study area

**Figure 1:**
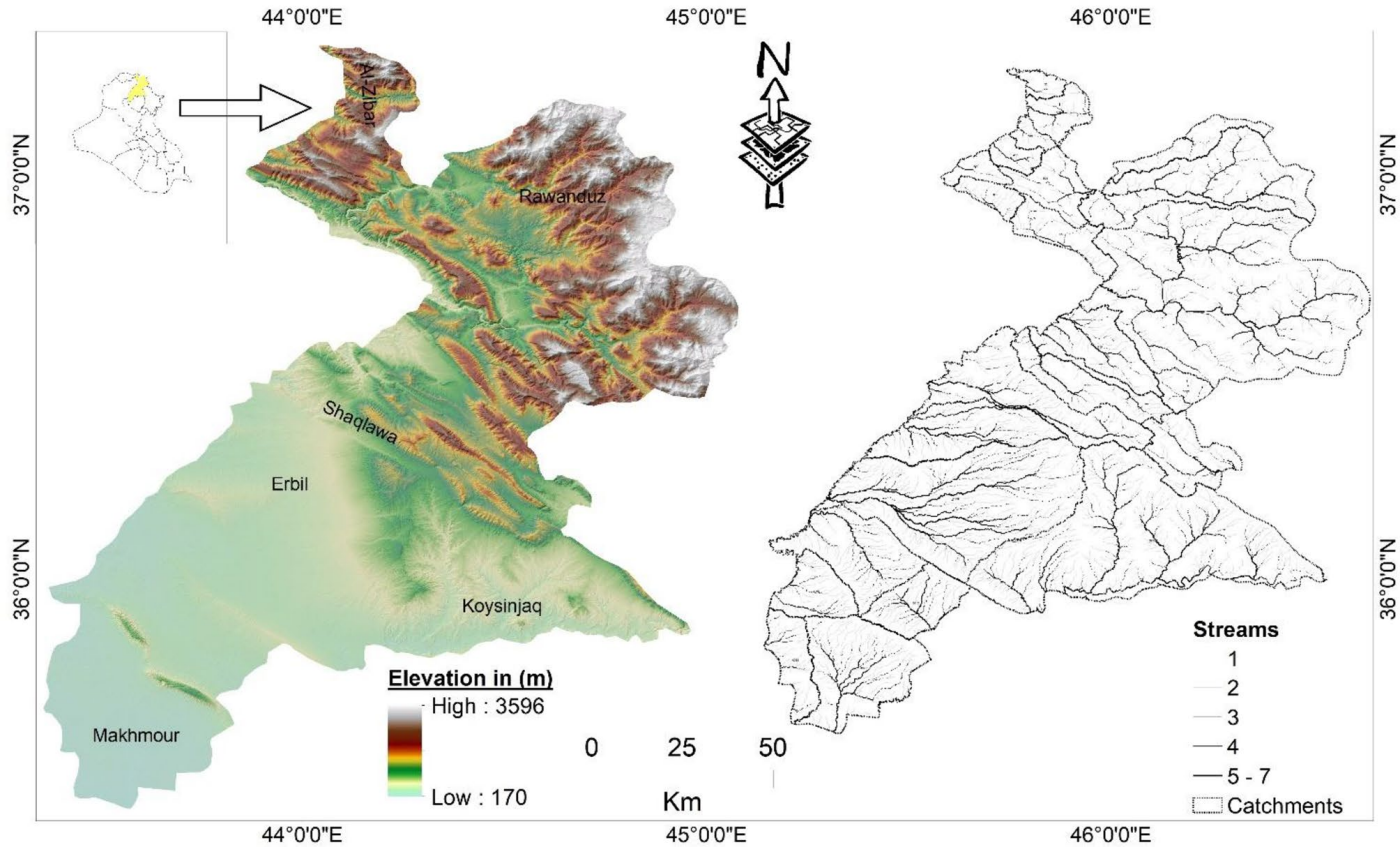
Location map of the study area.

The Kurdistan province, located in northern Iraq, was selected for this study (Fig. 1). The province, which is ~ 350 km from Baghdad, covers a total area of 1131.44 km^2^. It comprises five sub-districts: Erbil, Koysinjaq, Soran, Shaqlawa, and Choman. Topographically, the central regions are mostly flat, whereas the northeast and eastern regions are mostly mountainous and hilly. Erbil province has a semi-arid climate, with hot and dry summers (June to October) and cold and wet winters (December to March), with heavy rain from October to May. The annual rainfall fluctuates within the study area because of topographic variation, indicating a high potential for flash flood events. Annual rainfall in the northern regions is 1275 mm, while it is 612 mm in the southern regions. In general, the average temperature ranges from 6 to 16 °C in the winter and from 25 to 40 °C in the summer.

### Data analysis

**Figure 2:**
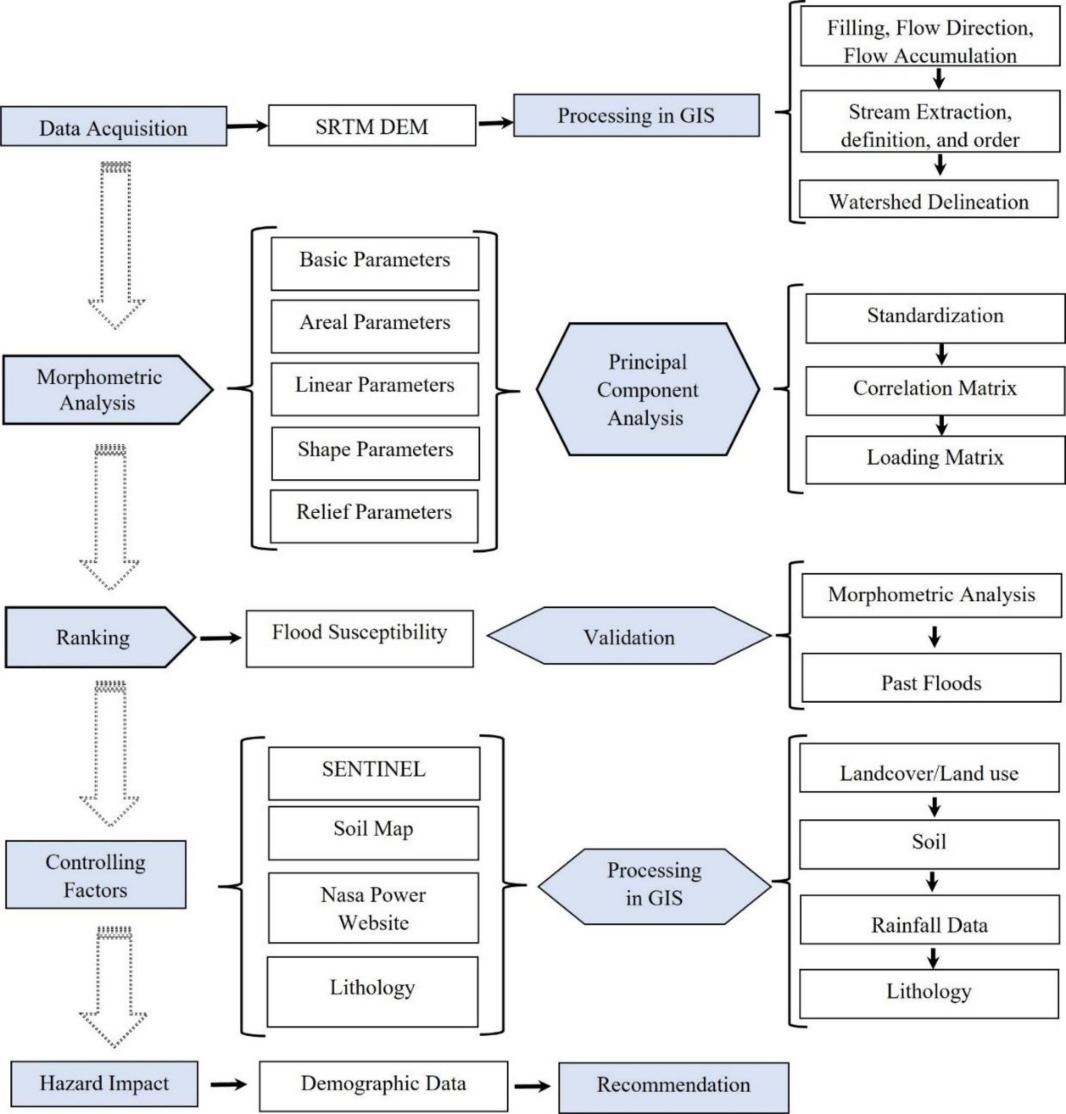
Flow chart showing the methodology applied in this study.

In this study, different data sources and methodologies are applied for identifying the most vulnerable flood areas (Fig. 2). The digital elevation model (DEM) from the Shuttle Radar Topographic Mission (SRTM) was used to extract streams and drainage basins from the study area. The SRTM is available for download from NASA’s Earth Explorer website (https://earthexplorer.usgs.gov/). It is a single-pass synthetic aperture radar interferometry (InSAR) campaign that provides unique DEM data with a resolution of 90 m. In contrast to the 10-m mesh DEM developed by the Geographical Survey Institute of Japan, the elevation error of the DEM ranges from + 7.4 m (forest areas) to 0.7 m (bare land), whereas the horizontal inaccuracy and shifting range from 0.13 arc-seconds for East/West to 0.19 arc-seconds for North/South. The high-resolution, open, accurate, comparable, and timely land use/land cover maps are derived from the ESRI 2020 Landcover database (https://livingatlas.arcgis.com/landcover/). The database is a global product for a user-specified area of interest and time period (2018–2022). The database was developed in conjunction with ESRI by Microsoft Planetary Computer and scaled using Microsoft Azure Batch using a deep learning model trained on 5 billion manually annotated Sentinel-2 pixels spread across 20,000 sample points worldwide. The final output comprises a composite of nine classes of LULC predictions over the required time period, with an overall accuracy of 86% worldwide, according to the validation points. In addition to the available geological map from The Iraq Geological Survey (http://en.geosurviraq.iq/), the soil map (1:50,000) was downloaded from the State Board for Land Reclamation, Ministry of Agriculture, Iraq (http://zeraa.gov.iq/). Soil classification has been developed using the FAO/UNEP international standard system based on the world soil map (FAO/UNESCO, 1974). Annual rainfall data from 1981 to 2022 are available from NASA’s Prediction of Worldwide Energy Resources (https://power.larc.nasa.gov). The service offers precipitation data with a high resolution that was produced from the Integrated Multi-satellite Retrievals for the GPM component of NASA’s Global Precipitation Measurement (GPM) program (IMERG). Global 0.1° × 0.1° latitude/longitude grids are used to resolve IMERG precipitation data. Rainfall data had a higher resolution than previous rainfall grid maps; thus, it was utilized to create a spatial rainfall distribution map. Additionally, annual rainfall maxima collected from the RainSphere database of CHRS at University of California, Irvine (https://rainsphere.eng.uci.edu/) for the period from 1983–2021 has been used for the frequency analysis.

The Weibull distribution is commonly used for frequency analysis as well as risk and reliability analysis of the lifetimes of systems and their components. Its applications have been reported frequently in hydrology and meteorology. Weibull in 1939 introduced the equation for the analysis of food magnitude for the corresponding return periods. Plotting the distribution involves ranking the data. It is specifically used with the exponential distribution and the Rayleigh distribution. In addition to other forms of probability distributions, this technique’s probability distribution is tied to the scale and shape parameters. The density function for this method is highly sensitive to changes in the value of the form parameter. Here, the rank of the food discharge for a particular distribution, the quantity of observations (N), and the frequency of occurrence (f), sometimes referred to as the probability percentage.

Moreover, historical data on the flood disaster was available through the OFDA/CRED International Disaster Database (EM-DAT), which provides crucial core information on the occurrence and consequences of 22,000 mass disasters that have occurred worldwide from 1900 to the present. The database is compiled from various sources, including non-governmental organizations, insurance providers, and academic institutions. EM-DAT is available to the general public at https://www.emdat.be/. For this study, historical losses including human and economic damages recorded in EM-DAT across all flood events from 2006 through 2021 in the study area to validate the resultant final map.

The ArcGIS hydrology spatial analyst tool extracts streams and delineates watersheds in a sequential manner during subsequent processing, beginning with filling sinks to remove any errors from the raw DEM, identifying the flow direction from higher to lower, assigning direction codes, and delineating the flow accumulation to determine the higher possibility flow accumulation pixels. The drainage network was built through a trial-and-error method, taking into account pixels with flow accumulation greater than a threshold of 900 m^2^. The user provided pour points, which were used to build the stream network and identify the sites with the highest flow accumulation. Pour locations were determined assuming that the calculated drainage basins encompassed the entire river basins. A stream order was determined using a stream ordering method^35,36^. Strahler’s ordering scheme places streams without tributaries in the first order, streams with two first-order tributaries in the second order, and so on. A well-drained basin comprises fifth-order stream channels^35^.

To assess flood susceptibility, 24 morphometric variables (linear, areal, shape, and relief) were selected from the study area and computed employing an established formula (Table 1). Stream order (No), stream number (Nu), stream length (Lu), sinuosity ratio (Si), and length of overland flow (Lo) were calculated and ranked as linear drainage morphometric criteria. Drainage density (Dd), drainage frequency (Fs), stream texture (Dt), constant of channel maintenance (C), and infiltration ratio (If) were also extracted and included as areal drainage aspects. Shape parameters calculated include the elongation ratio (Re), circulatory ratio (Rc), compactness coefficient (Cc), form factor (Ff), and shape index (Sw), whereas relief parameters include the basin relief (Bh), relief ratio (Rr), and ruggedness number (Rn). PCA represents a multivariate statistical method commonly and efficiently used to reduce data size while preserving as many changes as possible in the dataset for faster data processing^37^. Many variables can be reduced to significant factors using PCA, and the final factors summarize the original data. XLSTAT software was used to perform PCA for 24 morphometric variables in 24 watersheds. Because the approach is heavily reliant on the sum variance of the original variables, it is critical to represent the variables in a standard form, implying the same unit of measurement for each variable, to ensure a sample variance equal to 1. The correlation matrix is then examined with the total variance set to n. The inter-correlation matrix is obtained by standardizing the factors based on the following formula:

1 $$X = \left( {x_{ij} - x_{j} } \right)/S_{j}$$

where X denotes the matrix of standardized parameters; *x*_*ij*_ denotes the *i*th observation on the *j*th parameters; *i* = 1…N (Number of observation); *j* = 1…P (Number of observations); x_*j*_ denotes the *j*th parameters; and S*j* denotes the standard deviation of the *j*th parameters. The correlation matrix of parameters is the minor product moment of the standardized predictor measures divided by N and is given as follows:

2 $$R = \left( {x^{\prime} \times x} \right)$$

where, ${x}^{\prime}$ denotes the transpose of the standardized matrix of predictor parameters. The principal component loading matrix, which indicates how much an individual parameter is correlated with different factors, is obtained by pre-multiplying the characteristics vector with the square root of the characteristic values of the correlation matrix. Thus,

3 $$A = Q \times D^{0.5}$$

where A denotes the principal component loading matrix; Q denotes the characteristics vector of the correlation matrix; and D denotes the characteristics value of the correlation matrix. A rank (y) was determined for each basin using a relative ranking method based on the degree of flood susceptibility, where 1 indicates a very low susceptibility and 5 is a very high susceptibility in proportion to the value of each morphometric parameter (x). A linear interpolation method described by^38^ was used to calculate ranks. When the morphometric variable and the flood event have a positive correlation, Eq. (4) is used. Otherwise, Eq. (5) is used.

4 $$y \propto x, \;y^{\prime}n = \left( {y_{2} + y_{1} } \right)\left( {x^{\prime}_{n} - x_{\min } } \right)/\left( {x_{\max } - x_{\min } } \right) + y_{1}$$

5 $$y \propto 1/x,~\;y^{\prime } n = \left( {y_{2} + y_{1} } \right)\left( {x^{\prime } _{n} - ~x_{{\max }} } \right)/\left( {x_{{\min }} - x_{{\max }} } \right) + y_{1}$$

**Table 1:** List of parameters, formulas, and references used in the present study.

Parameter	Formula	Reference
Basin area (km)	*A*	Schumm (1956)
Basin perimeter (km)	*P*	Schumm (1956)
Basin length (km)	*L*_*b*_	Gregory and Walling (1973)
Total stream length (km)	*Lu* = *L1* + *L2 … L*_*u*_	Strahler (1957, 1964)
Total stream number	*Nu* = *N1* + *N2……N*_*u*_	Strahler (1957, 1964)
Drainage density	*Dd* = *L*_*u*_*/A*	Horton (1932)
Frequency	*Fs* = *∑Nn/A*	Horton (1932)
Elongation ratio	*Eb* = *√2 Ab∕lb*	Schumm (1956)
Circularity ratio	*C* = *4πA/P2*	Miller (1953)
Form factor	*F* = *A/L2*	Horton (1932)
Compactness coefficient	*c* = *P∕2√pA*	Gravel us (1914)
Sinuosity ratio	*SI* = *OL/EL*	Schumn (1956)
Longest channel	*Lc*	Horton (1932)
Slope	*S* = *Bh/Lb*	Schumn (1956)
Time of concentration	*Tc* = *0.0078L*_*b*_^*0.77*^*S*^*−0.38*^	Kirprich 1940
Length of overland flow	*Lo* = *0.5* × *1/Dd*	Schumm (1956)
Constant of channel maintenance	*C* = *1/Dd*	Schumn (1956)
Shape index	*L*_*b*_^*2*^*/A*	Horton (1945)
Infiltration number	*Fs* × *Dd*	Faniran 1986
Basin width	*Wb*	Horton (1932)
Drainage texture	*Td* = *∑N*_*u*_*/P*	Horton (1945)
Basin relief	*B*_*h*_ = *H − h*	Schumn (1956)
Relief ratio	*Rr* = *Hr/L*	Schumn (1956)
Ruggedness number	*Rn* = *H/Dd*	Strahler (1957, 1964)
Bifurcation ratio	*(Rb) Rb* = *Nn − 1/Nn*	Schumm (1956)

The flood risk degree increases with the parameter value for group 1 (Eq. 4), whereas the flood risk degree decreases with the parameter value for group 2 (Eq. 5). Finally, the number of basins was determined by adding the standardized scores for each attribute. The calculated data were used to classify the basins into groups based on the risk and degree of flood vulnerability.

Regardless of the method used for validation, it is essential to validate the flood susceptibility maps^39^. Morphometric parameters such as Rb, Dd, and Fs were usually compared to the proposed model in order to validate the use of the flood susceptibility map^16,40–42^. In this regard, basin divided into three zones of the vulnerable flood basins based on the Rb in conjunction with Dd and Fs^41^. Zone “A” represents low flood susceptibility, zone “B” represents a high-flood risk, and zone “C” represents an intermediate flood risk. Furthermore, known flood areas from past years were used to validate the final flood susceptibility map. In the present study, the same morphometric parameters (Rb, Dd, and Fs) of the drainage basins are computed and graphically presented for comparing and validating the obtained results. For additional validation, the past flood locations from the International Disaster Database (EM-DAT) are compared to the identified vulnerable areas.

## Results

### Morphometric analysis

**Figure 3:**
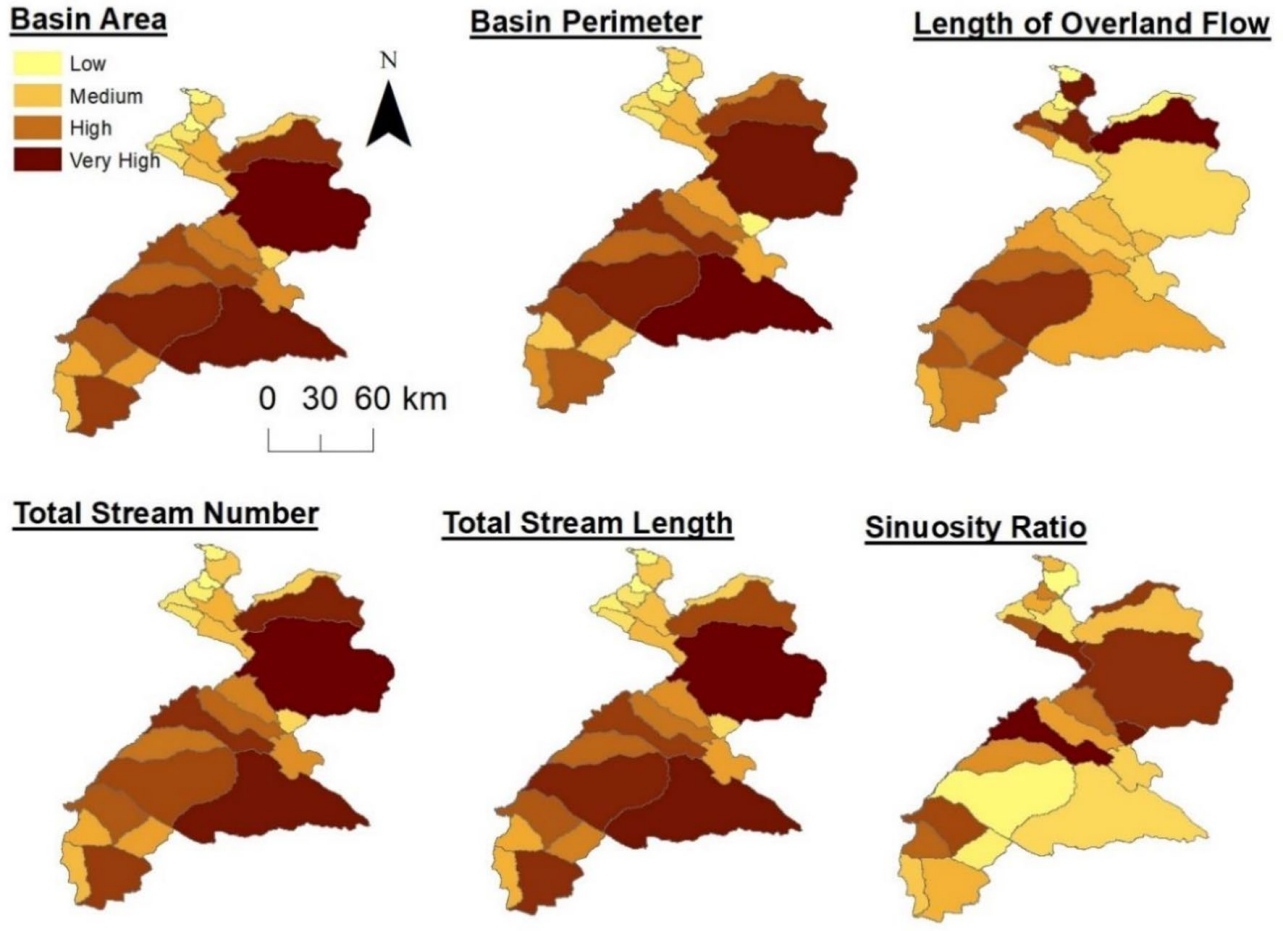
Spatial distribution of some morphometric parameters throughout the study area.

The peak discharge of the river, which causes flash floods, is significantly influenced by the geomorphometric features of the basins. For the 24 sub-basins (see supplementary, Table S1), the basin area (A) ranges from 4618 km^2^ in the central regions (basin no. 14) to 107.61 km^2^ in the northern regions (basin no. 20). In addition, basin perimeter (P) ranged from 57.71 km^2^ for basin no. 21 to 338.49 km^2^ for basin no. 14. The number of streams is relatively high for the central basins, such as 3, 4, 9, 14, and 16, indicating a significant discharge capability and flooding susceptibility. Among the 24 watersheds, 11 are assigned as fourth-order basins, 9 are fifth-order, 4 are sixth order, and 1 represents seventh order (see supplementary, Table S1). Further, the stream length characteristics of the different watersheds exhibited a noticeable variation, with basin no. 14 having the highest (2048 km) and basin no. 21 having the lowest (89 km) total length (Fig. 3). The overland flow (L_O_) significantly impacts the physiographical, topographical, and hydrological development of these streams in the drainage basins. The sinuosity index (Si) ranges from 0.54 to 1.41. risk of erosion^43^.

Because Dd has a direct impact on the flood hydrograph, susceptibility modeling heavily relies on it as a geomorphometric factor. The results of this study showed a variation between the upper basins in the central (highest values) and southern areas (lowest values), owing to variations in the slope, soil infiltration, and rainfall throughout the study area (Fig. 3). The higher infiltration numbers in the central and southern basins indicate lower infiltration and higher drainage conditions. The reciprocal of drainage density was used^44^ to define the morphological property of drainage basins regarding a constant of channel maintenance (C). Similarly, the Fs values of the studied basins range from 0.06 to 3.97 (see supplementary, Table S2). Such considerable values in the central areas imply moderate to high runoff and substantial peak discharge, posing flash flood risks compared to other basins within the study area (see supplementary, Figure S1). Furthermore, the Dt values vary from 0.38 to 8.95, with high values indicating very coarse texture as well as the presence of unstable slope materials, soft rocks, and an increased the calculated circularity ratios (Rc) for basins are between 0.19 (basin no. 9) and 0.62 (basin no. 13). Moreover, the elongation ratio (Re) of the basins ranges from 0.27 to 1.0, indicating that most watersheds are circular, with a few exceptions being elongated. Additionally, the shape factors for the basins range from 0.25 to 15.74, confirming their circular nature and making them more susceptible to flooding and experiencing greater erosion and sediment transport capacities.

Furthermore, the compactness coefficient (Cc) and form factor (Ff) are both used to determine the relationship between the hydrological conditions and the circular shape of the drainage basin. All watersheds have a Cc of ≥ 1, indicating less elongation, high erosion, and high flood susceptibility^45^. Horton (1945) introduced Ff to forecast the flow intensity of a drainage basin manipulating the direct relationship between peak discharge and the inverse relationship between the squares of the axial length of the drainage basin. Ff values for the basins range from 0.06 to 3.7. Watersheds with high Ff values experience high peak flows with short duration. In contrast, a long watershed with a low form factor experiences modest peak flows that last longer. The period of concentration (Tc), a measure of the physical properties of a basin, is often low in the central and southern basins (see Supplementary, Figure S2), supporting the latter’s high vulnerability to flooding. Basin relief (Bh) influences stream gradients, flooding patterns, and the number of transported sediments. Basins 1 and 2 have the lowest basin relief (50 m), whereas basin no. 14 has the highest (2770 m). In the study area, steep slopes dominated the northern and central basins, while gentle slopes dominated the southern basins (see supplementary, Table S2).

### Selection of the causative factors

The correlation matrix of 24 geomorphic parameters indicates four classes (see Supplementary, Table S3): First, excellent correlations were found between basin perimeter and basin area, total stream number and basin area, stream frequency and density, channel maintenance and stream frequency, infiltration number, drainage density and frequency, channel maintenance, Lo, and ruggedness number. Second, a significant correlation (correlation coefficient ˃0.75) was noted between total stream number and basin length, total stream number and total stream length, total stream length and perimeter of the basin, slope and form factor, ruggedness number and drainage density, stream frequency, as well as Lo and time of concentration. Third, a moderate correlation (correlation coefficient ˃0.60) existed between basin length and perimeter, total stream length and basin length, length of the main channel and total stream length, basin width and basin area, drainage texture and drainage density, length of overland flow, channel maintenance, relative relief ratio and compactness coefficient, as well as slope and elongation ratio. In addition, a weak correlation (correlation coefficient ˃0.7) existed between the time of concentration and total stream number, basin width and form factor, basin width and infiltration number, drainage texture and total stream number, and ruggedness number and basin width. At this stage, it is challenging to classify the parameters and determine the significance of the various aspects. Therefore, the PCA was used. The component loading matrix derived from the correlation matrix shows that the first four components, whose eigenvalues are greater than 1, account for about 85.3% of the total explained variance (Table 2). The rotated component matrix indicates that components are significant. Component 1 (areal parameters) is highly correlated with density, frequency, length of overland flow, constant of maintenance, infiltration number, and raggedness number, whereas component 2 (F2) is strongly correlated with the basin area, perimeter, basin length, total stream length and number, drainage density, main channel length, and time of concentration (Table 3).

**Table 2:** Total variance explained of the studied basins.

	Eigen value	Initial		Extracted	
		Variability (%)	Cumulative (%)	Variability (%)	Cumulative (%)
F1	8.47	35.27	35.27	35.14	35.14
F2	7.69	32.02	67.29	32.12	67.26
F3	2.69	11.22	78.51		
F4	1.62	6.76	85.27		
F5	0.78	3.24	88.51		
F6	0.44	1.85	90.36		
F7	0.15	0.64	90.99		
F8	0.1	0.41	91.41		
F9	0.06	0.25	91.65		
F10	0.04	0.15	91.80		
F11	0.01	0.04	91.84

**Table 3:** Rotated component matrix of the geomorphic parameters.

Parameter	D1	D2
A	− 0.25	0.85
P	− 0.24	0.91
L_b_	0.31	0.85
Lu	0.01	0.9
Nu	0.27	0.92
Dd	0.95	0.08
Fs	0.92	− 0.19
Bh	− 0.07	− 0.66
Er	− 0.76	− 0.05
Cr	− 0.24	− 0.18
Ff	− 0.69	− 0.9
Cc	0.5	− 0.79
Sr	− 0.22	0.18
Lc	0.34	0.85
S	− 0.24	0.56
Rr	0.39	− 0.66
Tc	0.1	0.78
Lo	0.95	0.08
Cc	0.95	0.08
Si	− 0.59	0.02
If	0.88	− 0.03
Bw	− 0.61	0.55
Dr	0.79	0.49
Rn	0.86	− 0.01

As previously stated, the most crucial morphometric characteristics exhibit high correlation. As a result, only twelve of the twenty-four parameters used in this study are used to map flood susceptibility. Thus, the PCA was useful in removing the variables with the least relevance and grouping the remaining variables into physically significant components.

### Flood susceptibility mapping

**Figure 4:**
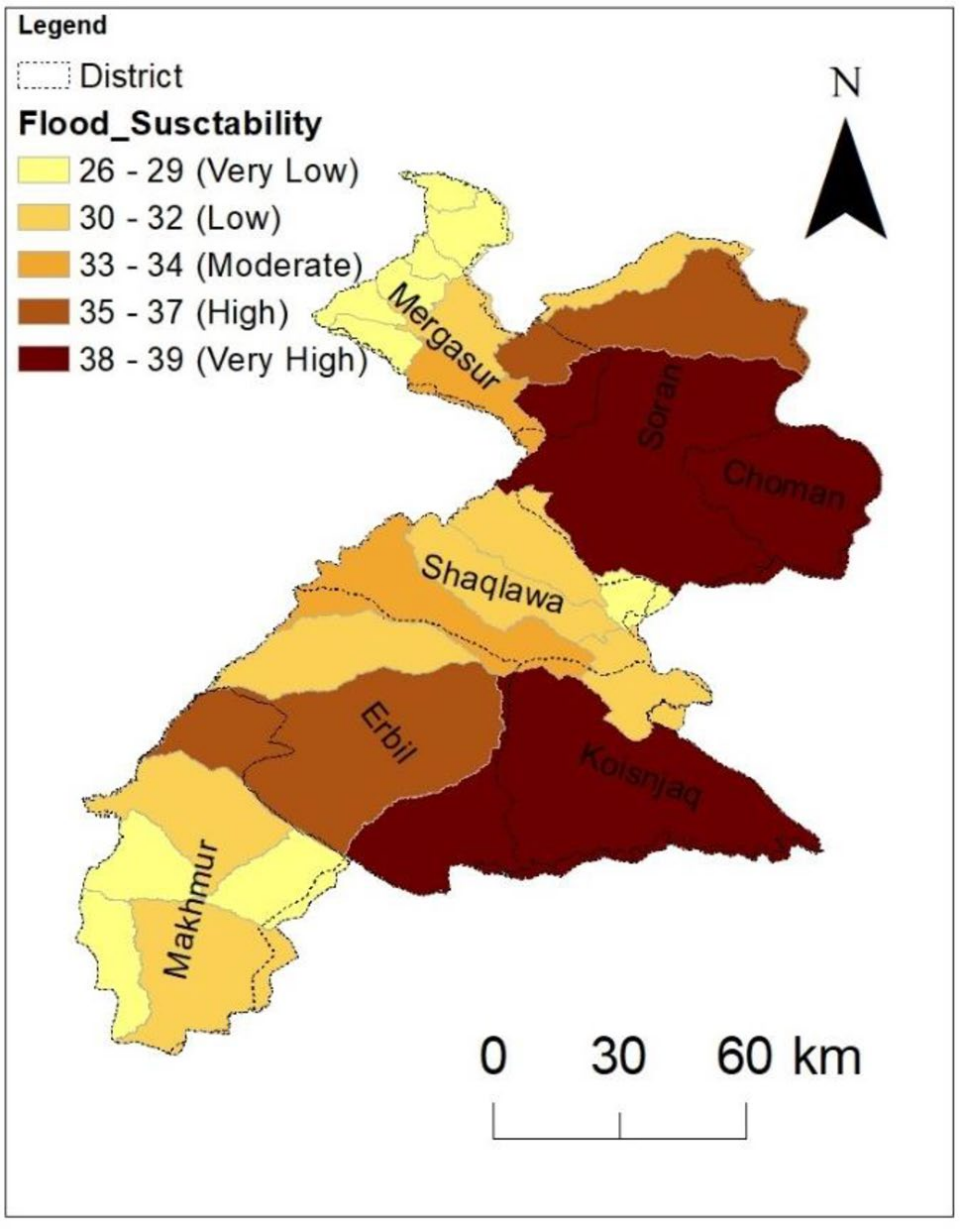
Flood susceptibility map and susceptible areas in the different districts.

The degree of flash flood susceptibility is determined for 24 basins using the geomorphometric ranking approach (see Supplementary, Table S4). The approach has been applied by many researchers to evaluate the impacts of the morphometric characteristics of the drainage basin on flood susceptibility^14,46^. The method was used to calculate the cumulative ranking score for various geomorphometric features divided into two groups: positively correlated features (total stream number and length, basin area, drainage density, frequency and texture, and ruggedness number) and negatively correlated features (basin length, length of overland flow, time of concentration, stream maintenance, and infiltration number). The cumulative ranking score was used to divide the study area into susceptibility zones using geomorphometric data. The geomorphometric number is inversely proportional to the flash flood susceptibility of basins. According to the investigation, all basins have a geomorphometric number between 26 and 39. Out of the 24 basins in the present study, basins 16, 3, and 14 have the highest geomorphometric values (36–39), signifying the zone most vulnerable to flash floods and making up a maximum land area of 38.58% of the study area. The moderately susceptible basins are basins 4, 8, 9, 10, 12, and 15, which cover a land area of 27.86% and have geomorphometric numbers between 30 and 35. The remaining basins comprise 33.47% of the study; they have limited exposure to floods and have geomorphometric numbers < 30 (Fig. 4).

**Figure 5:**
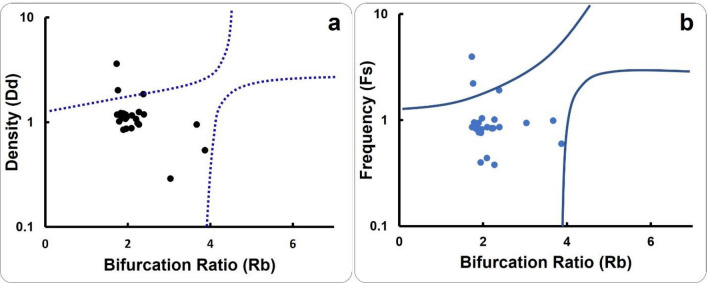
validating the flash flood hazard degree using (**a**) Rb versus Dd; (**b**) Rb versus Fs.

It is critical to validate flood susceptibility to illustrate how effectively the map performed. Rb is considered to represent the most important parameter for determining the degree of risk and has a greater impact than the other parameters^41^. Rb versus Dd and Fs relationships were displayed in Fig. 5. This method differs from PCA and ranking methods, which equalize the impacts of all parameters and are screened to identify the most significant factors. This difference can be attributed to the various weights that are assigned to the various parameters.

## Discussions

**Figure 6:**
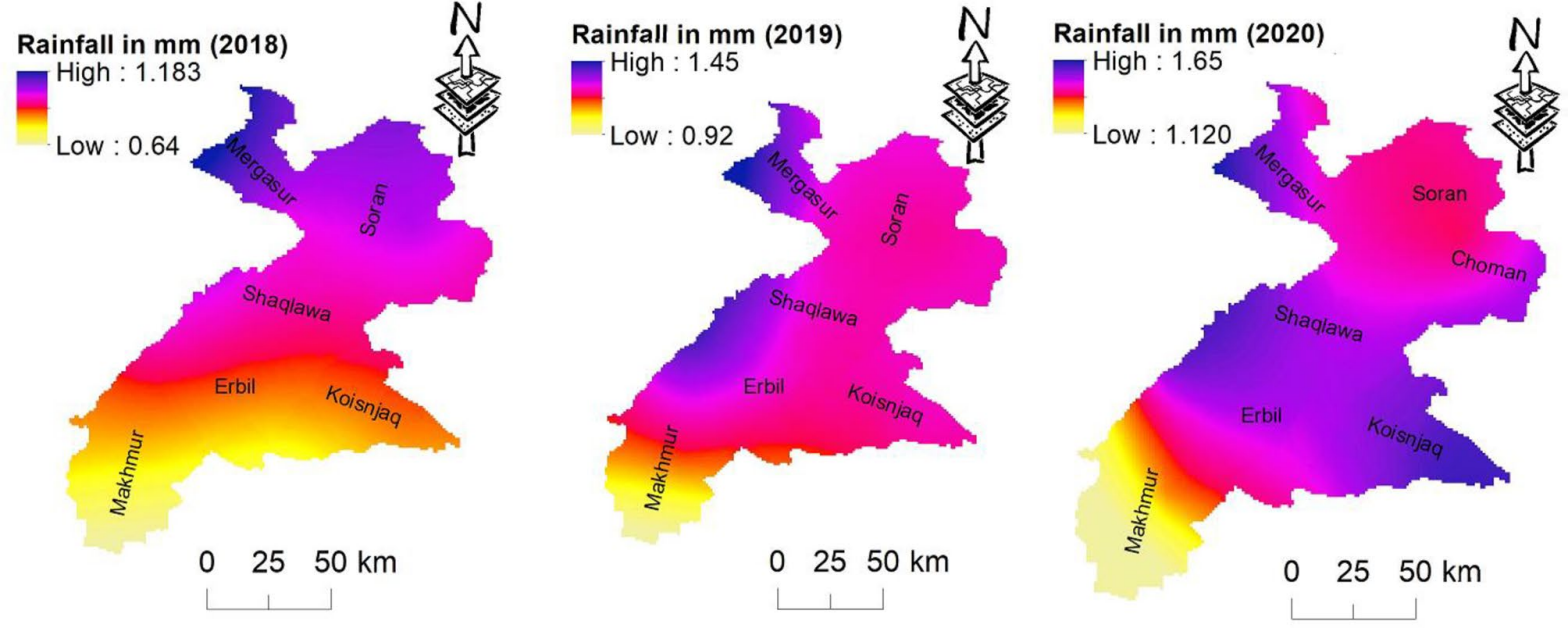
Annual rainfall distribution for the period 2018–2020.

In arid regions, flash floods are among the most devastating natural disasters, responsible for loss of life and serious damage to vital infrastructure and buildings^47^. Reliable and accurate data on flash flooding in arid environments are frequently lacking due to the remoteness and sparse habitation of such areas^21^. Delineation of flood susceptible areas is considered the basic action toward flood mitigation. In this study, the flood-prone areas are delineated through the integration of flood causative factors in a GIS environment, PCA, and ranking method. Results of the PCA indicate that the hydro-morphometric characteristics of the drainage basins can be combined into two main groups that account for 85% of the variation of the data and have clear physical meaning. The PC represents drainage density, stream frequency, length of overland flow, and constant maintenance as the most important predictors of flash flooding, which is consistent with what is commonly believed. Consequently, basins of the eastern areas are more susceptible to flooding when compared to other basins throughout the study area. Typically, basins in these areas are characterized by a high drainage density, high stream frequency, and high overland flow indicating high runoff potentiality and less infiltration^48,49^. In addition to geomorphic features, the duration of rainfall, soil erosion, increased population, urbanization and settlements, increased infrastructure, the efficiency of the sewer system, and public awareness, could contribute to flash floods^50^. In recent years, Climate change including variability in temperature, rainfall patterns, and storm activity is increasing^51^. There is rising evidence that the global climate is changing in response to natural and human factors^52^. Spatial distribution of the average annual rainfall indicates a variation from the western to the eastern parts of the study area (Fig. 6). Six heavy rainfall events were recorded between 2008 and 2018^53^.

Over the study area, intense rainfall has returned almost every two years, causing numerous flash floods with no warning. Results show that the total annual rainfall of the study area ranges from 244 to 633 mm, with an average of 436 mm (see Supplementary, Figure S3). The maximum annual daily rainfall varies from 12 to 45 mm with an average of 25 mm. In addition, the number of rainy days ranges between 111 to 234 mm, with an average of about 194 days/year. Based on the maximum daily rainfall data, statistical investigation is accomplished through the Weibull method and examined by probability distribution to fit the rainfall data to reach the best distribution curve, which matches the actual data of each rainfall station. From this graph, it is evident that that in the following periods (5, 10, and a 100 years), an increase in the rainfall estimates is predicted (see Supplementary, Figure S3).

Moreover, changes in land use/cover significantly affect the occurrence of surface water, infiltration, evapotranspiration, and soil erosion^54,55^. Evidently, the most common change that occurs in this area represents a transition from earlier natural to impervious surfaces. As cities expand due to population growth, fewer wetlands and more built-up regions exist. Infrastructure in populated regions is associated with an increase in impervious surface, which changes the hydrologic characteristics of the area (see supplementary, Figure S4). Therefore, soil infiltration decreases, surface runoff increases, and more sediment loads are transported into water bodies downstream, contributing to flooding. Thus, changes in land use and land cover cause soil compaction, reducing soil infiltration, and increasing runoff volume and speed^56^. The sewer system in the study area is old, disconnected, and insufficient to reduce the effectiveness of runoff during flood events. During the most intense rainfall events, outflow occurred due to sewer system failure. Finally, poor sewer system management and a lack of public awareness comprise factors that contribute to flash floods in this area. For proper flood management, clean and efficient inlets and sewer lines are essential.

A significant outcome of the present study is a regional flood susceptibility map of an arid and vulnerable climate area, which is one of the most flood-affected regions of the country. The value of the current work was expected to be limited by the large basin characteristics, which, in turn, could impact the expectation of the results to differ on a smaller scale. Interestingly, a comparison of the regional study results (here is the current study) with the results of local studies in the same area is beneficial. Therefore, this study shows that a large set of hydro-morphometric parameters can be reduced to a smaller set without loss of information, indicating redundancy in the data. The final map was certified for greater accuracy by comparing the flood hazard map with historical flood data from the International Disaster Database (EM-DAT), which highlights flood-prone locations (Table 4). Floods caused by heavy rain have become more common in recent decades. The findings of this investigation are in excellent agreement with the EM-DAT flood data. Most of the recorded injuries and fatalities in the terrible flash flood events of 2006, 2009, 2011, and 2021 occurred in extremely vulnerable regions (Table 4). Additionally, the investigation showed that 44.56% of all human settlements are in extremely sensitive basins (Choman, Mergasur, Soran, and Erbil). It is expected that the prepared map will help disaster managers, planners, and local authorities to improve their understanding of flood vulnerability in the region. It can also be expected that the map will assist in guiding the operational responses of the various authorities in the region, especially in areas that have been identified as high-risk zones. The most effective strategy to lessen flooding is to implement a variety of carefully considered strategies that are suited to the specific catchment of interest^57^ assert that no single mitigation approach has ever been able to effectively address the flood problem. Consequently, they claimed a better strategy that incorporates land use planning, engineering fixes, flood preparedness, and emergency management in the affected lowlands. This strategy would also consider the social and economic needs of communities in both the highland, which are frequently source areas and the lowland.

**Table 4:** Past flood hazard level within the study area according to the EM-DAT Database.

	2006	2006	2008	2009	2011	2021
Location	Soran	Erbil	Choman	Mergasur	Erbil	Erbil
Total deaths	20.00		4.00	2.00	6.00	14.00
No injured	20.00				1.00	
No affected	18,000.00	41,890.00		3000.00		7500.00
No homeless			600.00		2000.00	
Total affected	18,020.00	41,890.00	600.00	3000.00	2001.00	7500.00
Total damages (‘000 US$)		1300.00				14,000.00
Total damages, adjusted (‘000 US$)		1747.00				14,000.00
CPI	74.40	74.40	79.46	79.17	83.01	100.00

While there is typically nothing that can be done to reduce the rainfall amount that a region receives, there are effective steps that can be taken to reduce the amount of surface runoff, which is a source of flooding. To mitigate the effects of the increased impervious surface caused by urban dwelling developments, rainwater harvesting is considered as an effective method for achieving this. The technique has been used in many developing countries with inadequate drainage systems as a means of mitigating some anthropogenically induced ecological problems^58–60^. Along with rainwater harvesting, there should be efficient land use/landcover management practices that could reduce the flood hydrograph peak which is usually of utmost concern. Reducing impermeable surfaces, unless essential, can also result in greater advantages. In order to effectively and sustainably reduce flooding, the use of materials and construction designs like permeable pavements for parking lots and sidewalks, green roofs, rain gardens, retention cisterns, and natural rehabilitation are recommended.

## Conclusions

In this study, an effective mitigation technique for achieving early flood warning and effective flood crisis management by accurately mapping flood susceptibility in the Erbil area of northern Iraq was implemented through the integration of geospatial techniques, watershed characteristics, and PCA, flood risk zones were identified. The PCA analysis reduced the initial set of 24 morphometric parameters to two important statistical components, using the component loading matrix, which provided insights into the correlations between the morphometric variables and helped to identify the key factors influencing flood susceptibility. Based on the morphometric relative ranking, the study categorized the drainage basins into three classes: low, moderate, and high flood susceptibility. The analysis revealed that three basins have a considerable degree of flood susceptibility, six basins have a moderate degree, and the remaining basins have a low degree. The produced susceptibility mapping was validated by comparison with historical flood occurrences in the study area. The observed correlation between the identified flood-susceptible areas and past flood damages, such as economic losses and human casualties, further supports the accuracy of the flood susceptibility mapping approach. The integrated methodology of morphometric analysis, PCA, and GIS can be recommended as a valuable tool for efficient planning, management, and decision-making to detect flood hazards, create flood mitigation plans, and enhance the resilience of the region to future flood risks. In contrast to other methods routinely used for creating susceptibility maps, the present methodology requires considerably simpler input data, notably morphometric data, which can be readily retrieved from accessible DEMs with good resolution. The proposed method does not require other parameters, such as an inventory map, which is time-consuming and expensive to build. To fully curb the problem of flooding, it is highly recommended to include artificial intelligence in predicting the flood phenomenon in the region because of its importance in determining future plans and preventing flood dangers in the region.

## Supplementary Information

Supplementary Information.

## Data Availability

The datasets used and/or analyzed during the current study are available from the corresponding author on reasonable request.
