# Supplementary material for: Flood susceptibility mapping utilizing the integration of geospatial and multivariate statistical analysis, Erbil area in Northern Iraq as a case study
Source: Sci Rep. 2023 Jul 24;13:11919. doi: 10.1038/s41598-023-39290-4 (PMC10366121; doi:10.1038/s41598-023-39290-4)

**Supplementary Materials**

**Flood susceptibility mapping utilizing the integration of geospatial and multivariate statistical Analysis, Erbil Area in Northern Iraq as a case study.**

Alaa Ahmed^1,2, 3*^, Ali Al Maliki^4^, Bassim Hashim^4^, Dalal Alshamsi^1,2^, Hasan Arman^1^ and Ahmed Gad^1,5^

1. Geosciences Department, United Arab Emirates University, Al Ain, 15551, United Arab Emirates.
2. National Water and Energy Center, United Arab Emirates University, Al Ain, 15551, United Arab Emirates.
3. Geology Department, Division of Water Resource, Desert Research Center, Mathaf El Matariya Street, Cairo, 11753, Egypt
4. Environment and Water Directorate, Ministry of Science and Technology, Baghdad 765, Iraq
5. Geology Department, Faculty of Science, Ain Shams University, Cairo 11566, Egypt

* Corresponding Author: E-mail address: aahmed83@uaeu.ac.ae

**Table S1** Basic and linear aspects of the drainage basins in the study area

|  | **Basic Parameters** | | | **Linear Aspects** | | | | |
| --- | --- | --- | --- | --- | --- | --- | --- | --- |
| Basin | **A** | **P** | **L_b_** | **N_o_** | **N_u_** | **L_u_** | **S_i_** | **L_O_** |
| **1** | 1187.41 | 208.23 | 44.31 | 6.00 | 1019.00 | 1373.01 | 1.07 | 0.58 |
| **2** | 533.39 | 113.86 | 19.42 | 5.00 | 410.00 | 648.00 | 1.03 | 0.61 |
| **3** | 3646.45 | 374.30 | 58.65 | 4.00 | 1450.00 | 3941.17 | 1.04 | 0.54 |
| **4** | 2680.76 | 294.74 | 77.62 | 6.00 | 1015.00 | 3343.31 | 0.95 | 0.62 |
| **5** | 926.82 | 209.03 | 40.82 | 6.00 | 798.00 | 1089.42 | 1.18 | 0.59 |
| **6** | 448.33 | 110.74 | 24.90 | 4.00 | 384.00 | 534.37 | 1.17 | 0.60 |
| **7** | 418.12 | 138.70 | 18.53 | 4.00 | 351.00 | 446.43 | 1.05 | 0.53 |
| **8** | 910.13 | 193.37 | 50.72 | 5.00 | 695.00 | 1073.10 | 1.13 | 0.59 |
| **9** | 1133.30 | 277.08 | 68.04 | 5.00 | 1023.00 | 1229.86 | 1.41 | 0.54 |
| **10** | 719.91 | 162.84 | 56.92 | 5.00 | 726.00 | 684.18 | 1.08 | 0.48 |
| **11** | 568.70 | 136.90 | 34.50 | 5.00 | 565.00 | 540.34 | 1.05 | 0.48 |
| **12** | 621.43 | 137.22 | 45.35 | 5.00 | 591.00 | 634.37 | 1.14 | 0.51 |
| **13** | 200.08 | 63.68 | 17.53 | 4.00 | 169.00 | 195.58 | 1.41 | 0.49 |
| **14** | 4618.10 | 338.49 | 88.02 | 7.00 | 2048.00 | 4073.10 | 1.33 | 0.44 |
| **15** | 403.53 | 131.21 | 40.94 | 5.00 | 329.00 | 349.66 | 1.37 | 0.43 |
| **16** | 333.42 | 147.95 | 75.26 | 5.00 | 1324.00 | 1205.33 | 1.06 | 1.81 |
| **17** | 430.87 | 128.99 | 45.43 | 5.00 | 260.00 | 232.74 | 1.20 | 0.27 |
| **18** | 185.09 | 68.86 | 26.78 | 4.00 | 356.00 | 342.87 | 1.04 | 0.93 |
| **19** | 158.43 | 71.44 | 22.59 | 4.00 | 164.00 | 176.22 | 1.17 | 0.56 |
| **20** | 107.61 | 57.71 | 15.70 | 4.00 | 156.00 | 131.15 | 1.04 | 0.61 |
| **21** | 296.72 | 94.58 | 14.08 | 5.00 | 89.00 | 86.29 | 1.13 | 0.15 |
| **22** | 126.12 | 72.34 | 19.86 | 5.00 | 280.00 | 254.21 | 0.54 | 1.01 |
| **23** | 1463.52 | 245.34 | 19.90 | 4.00 | 93.00 | 88.12 | 1.06 | 0.03 |
| **24** | 164.98 | 64.07 | 17.28 | 4.00 | 155.00 | 140.04 | 1.08 | 0.42 |

**Table S2** Areal, shape, and relief parameters of the drainage basins

|  | **Dd** | **Fs** | **Dt** | **C** | **If** | **Er** | **Cr** | **Cc** | **Ff** | **Sw** | **Br** | **S** | **Rn** | **Sr** |
| --- | --- | --- | --- | --- | --- | --- | --- | --- | --- | --- | --- | --- | --- | --- |
| **1** | 1.16 | 0.86 | 4.89 | 1.16 | 0.99 | 0.88 | 0.34 | 0.18 | 0.60 | 1.17 | 50.00 | 1.04 | 0.06 | 1.07 |
| **2** | 1.21 | 0.77 | 3.60 | 1.21 | 0.93 | 1.34 | 0.52 | 0.21 | 1.41 | 1.30 | 50.00 | 2.45 | 0.06 | 1.03 |
| **3** | 1.08 | 0.40 | 3.87 | 1.08 | 0.43 | 1.16 | 0.33 | 0.10 | 1.06 | 2.51 | 540.00 | 8.71 | 0.58 | 1.04 |
| **4** | 1.25 | 0.38 | 3.44 | 1.25 | 0.47 | 0.75 | 0.39 | 0.11 | 0.44 | 2.64 | 620.00 | 7.80 | 0.77 | 0.95 |
| **5** | 1.18 | 0.86 | 3.82 | 1.18 | 1.0 | 0.84 | 0.27 | 0.23 | 0.56 | 1.16 | 160.00 | 3.61 | 0.19 | 1.18 |
| **6** | 1.19 | 0.86 | 3.47 | 1.19 | 1.02 | 0.96 | 0.46 | 0.25 | 0.72 | 1.17 | 290.00 | 9.85 | 0.35 | 1.17 |
| **7** | 1.07 | 0.84 | 2.53 | 1.07 | 0.90 | 1.25 | 0.27 | 0.33 | 1.22 | 1.19 | 30.00 | 2.74 | 0.03 | 1.05 |
| **8** | 1.18 | 0.76 | 3.59 | 1.18 | 0.90 | 0.67 | 0.31 | 0.21 | 0.35 | 1.31 | 750.00 | 15.56 | 0.88 | 1.13 |
| **9** | 1.09 | 0.90 | 3.69 | 1.09 | 0.98 | 0.56 | 0.19 | 0.24 | 0.24 | 1.11 | 950.00 | 11.84 | 1.03 | 1.41 |
| **10** | 0.95 | 1.01 | 4.46 | 0.95 | 0.96 | 0.53 | 0.34 | 0.23 | 0.22 | 0.99 | 790.00 | 12.69 | 0.75 | 1.08 |
| **11** | 0.95 | 0.99 | 4.13 | 0.95 | 0.94 | 0.78 | 0.38 | 0.24 | 0.48 | 1.01 | 520.00 | 15.60 | 0.49 | 1.05 |
| **12** | 1.02 | 0.95 | 4.31 | 1.02 | 0.97 | 0.62 | 0.41 | 0.22 | 0.30 | 1.05 | 1250.00 | 26.85 | 1.28 | 1.14 |
| **13** | 0.98 | 0.84 | 2.65 | 0.98 | 0.83 | 0.91 | 0.62 | 0.32 | 0.65 | 1.18 | 430.00 | 19.80 | 0.42 | 1.41 |
| **14** | 0.88 | 0.44 | 6.05 | 0.88 | 0.39 | 0.87 | 0.51 | 0.07 | 0.60 | 2.25 | 2770.00 | 29.41 | 2.44 | 1.33 |
| **15** | 0.87 | 0.82 | 2.51 | 0.87 | 0.71 | 0.55 | 0.29 | 0.33 | 0.24 | 1.23 | 880.00 | 27.60 | 0.76 | 1.37 |
| **16** | 3.62 | 3.97 | 8.95 | 3.62 | 14.36 | 0.27 | 0.19 | 0.44 | 0.06 | 0.25 | 2560.00 | 36.66 | 9.25 | 1.06 |
| **17** | 0.54 | 0.60 | 2.02 | 0.54 | 0.33 | 0.52 | 0.33 | 0.30 | 0.21 | 1.66 | 2080.00 | 166.67 | 1.12 | 1.20 |
| **18** | 1.85 | 1.92 | 5.17 | 1.85 | 3.56 | 0.57 | 0.49 | 0.37 | 0.26 | 0.52 | 1500.00 | 47.18 | 2.78 | 1.04 |
| **19** | 1.11 | 1.04 | 2.30 | 1.11 | 1.15 | 0.63 | 0.39 | 0.45 | 0.31 | 0.97 | 1660.00 | 92.74 | 1.85 | 1.17 |
| **20** | 1.22 | 0.83 | 2.70 | 1.22 | 1.01 | 0.75 | 0.41 | 0.54 | 0.44 | 1.21 | 1470.00 | 79.50 | 1.79 | 1.04 |
| **21** | 0.29 | 0.94 | 0.94 | 0.29 | 0.27 | 1.38 | 0.42 | 0.32 | 1.50 | 1.06 | 1630.00 | 108.88 | 0.47 | 1.13 |
| **22** | 2.02 | 2.22 | 3.87 | 2.02 | 4.47 | 0.64 | 0.30 | 0.57 | 0.32 | 0.45 | 1530.00 | 44.17 | 3.08 | 0.54 |
| **23** | 0.06 | 0.06 | 0.38 | 0.06 | 0.00 | 2.17 | 0.31 | 0.17 | 3.70 | 15.74 | 1600.00 | 130.19 | 0.10 | 1.06 |
| **24** | 0.85 | 0.94 | 2.42 | 0.85 | 0.80 | 0.84 | 0.51 | 0.39 | 0.55 | 1.06 | 830.00 | 47.95 | 0.70 | 1.08 |

**Table S3** Inter-correlation matrix of the geomorphic parameters


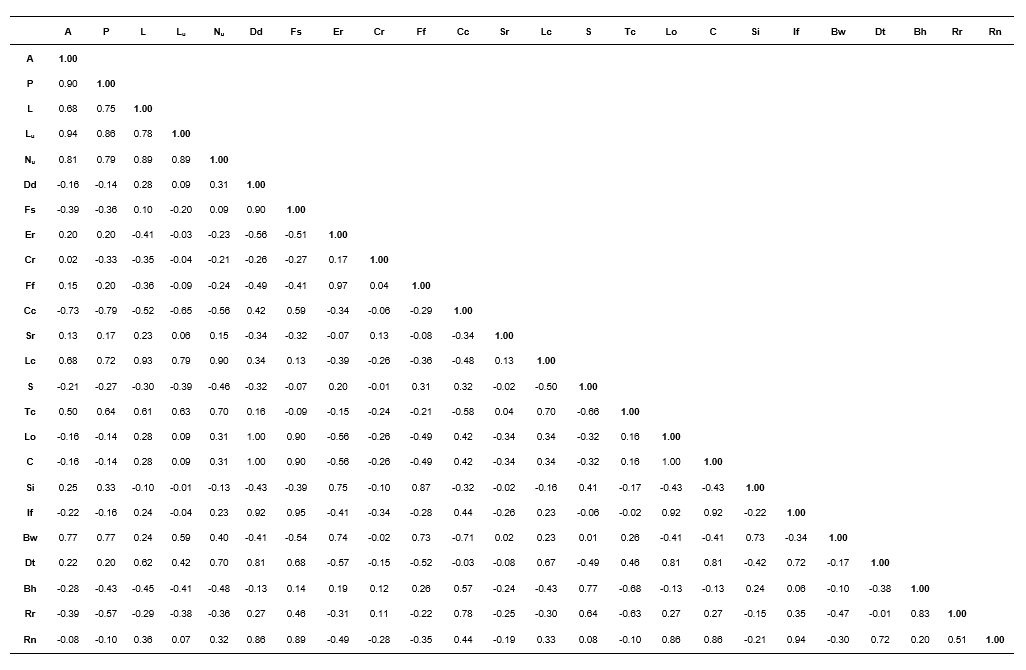


**Table S4** Ranking score and total score of the flood susceptibility for the different basins

|  | A | L_b_ | L_u_ | N_u_ | Dd | Fs | Tc | Lo | C | If | Dt | Rn | Rank |
| --- | --- | --- | --- | --- | --- | --- | --- | --- | --- | --- | --- | --- | --- |
| 1 | 2 | 3 | 2 | 3 | 1 | 1 | 1 | 4 | 4 | 5 | 3 | 1 | 29 |
| 2 | 1 | 1 | 2 | 2 | 1 | 1 | 4 | 4 | 4 | 5 | 3 | 1 | 27 |
| 3 | 4 | 3 | 5 | 4 | 1 | 0 | 3 | 4 | 4 | 5 | 3 | 1 | 37 |
| 4 | 3 | 4 | 4 | 3 | 1 | 0 | 2 | 4 | 4 | 5 | 2 | 1 | 35 |
| 5 | 2 | 2 | 2 | 2 | 1 | 1 | 3 | 4 | 4 | 5 | 3 | 1 | 29 |
| 6 | 1 | 2 | 1 | 2 | 1 | 1 | 4 | 4 | 4 | 5 | 2 | 1 | 28 |
| 7 | 1 | 1 | 1 | 2 | 1 | 1 | 4 | 4 | 4 | 5 | 2 | 1 | 27 |
| 8 | 2 | 3 | 2 | 2 | 1 | 1 | 4 | 4 | 4 | 5 | 3 | 1 | 31 |
| 9 | 2 | 4 | 2 | 3 | 1 | 1 | 3 | 4 | 4 | 5 | 3 | 1 | 32 |
| 10 | 2 | 3 | 2 | 2 | 1 | 1 | 3 | 4 | 4 | 5 | 3 | 1 | 31 |
| 11 | 2 | 2 | 1 | 2 | 1 | 1 | 4 | 4 | 4 | 5 | 3 | 1 | 30 |
| 12 | 1 | 3 | 2 | 2 | 1 | 1 | 4 | 4 | 4 | 5 | 3 | 2 | 31 |
| 13 | 1 | 1 | 1 | 1 | 1 | 1 | 4 | 4 | 4 | 5 | 2 | 1 | 27 |
| 14 | 1 | 5 | 5 | 5 | 1 | 0 | 3 | 4 | 4 | 5 | 4 | 2 | 39 |
| 15 | 5 | 2 | 1 | 1 | 1 | 1 | 4 | 4 | 4 | 5 | 2 | 1 | 33 |
| 16 | 1 | 4 | 2 | 4 | 4 | 4 | 4 | 1 | 1 | 1 | 5 | 5 | 36 |
| 17 | 1 | 3 | 1 | 1 | 1 | 1 | 5 | 4 | 4 | 5 | 2 | 1 | 30 |
| 18 | 1 | 2 | 1 | 2 | 2 | 2 | 5 | 3 | 3 | 4 | 3 | 2 | 29 |
| 19 | 1 | 1 | 1 | 1 | 1 | 1 | 5 | 4 | 4 | 5 | 2 | 2 | 28 |
| 20 | 1 | 1 | 1 | 1 | 1 | 1 | 5 | 4 | 4 | 5 | 2 | 2 | 27 |
| 21 | 1 | 1 | 1 | 1 | 0 | 1 | 5 | 5 | 5 | 5 | 1 | 1 | 27 |
| 22 | 1 | 1 | 1 | 1 | 2 | 2 | 4 | 3 | 3 | 4 | 3 | 2 | 28 |
| 23 | 1 | 1 | 1 | 1 | 0 | 0 | 5 | 5 | 5 | 5 | 1 | 1 | 26 |
| 24 | 2 | 1 | 1 | 1 | 1 | 1 | 5 | 4 | 4 | 5 | 2 | 1 | 29 |

**Fig. S1** Spatial analysis of the morphometric analysis throughout the study area


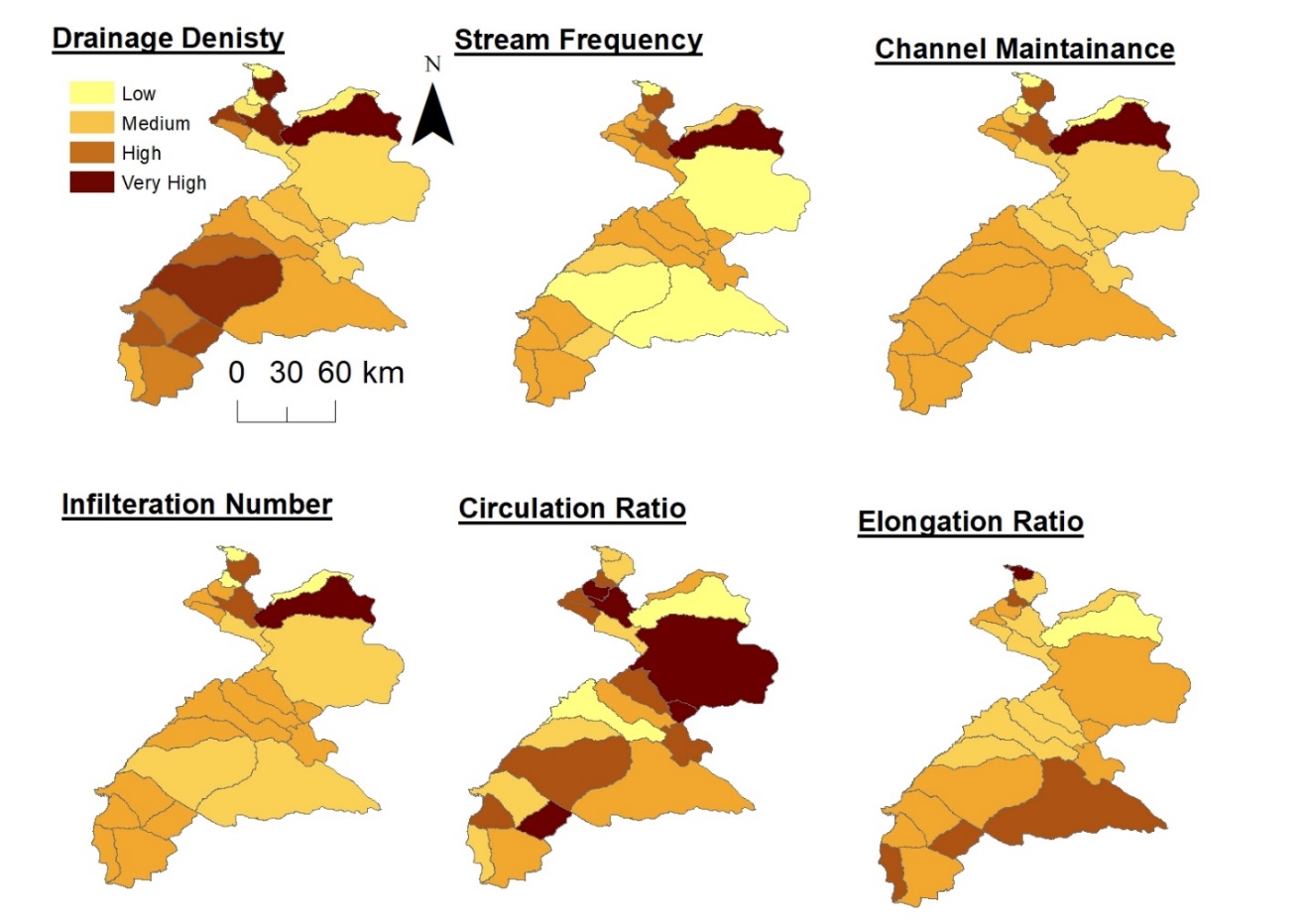


**Fig. S2** Spatial analysis of the morphometric analysis throughout the study area


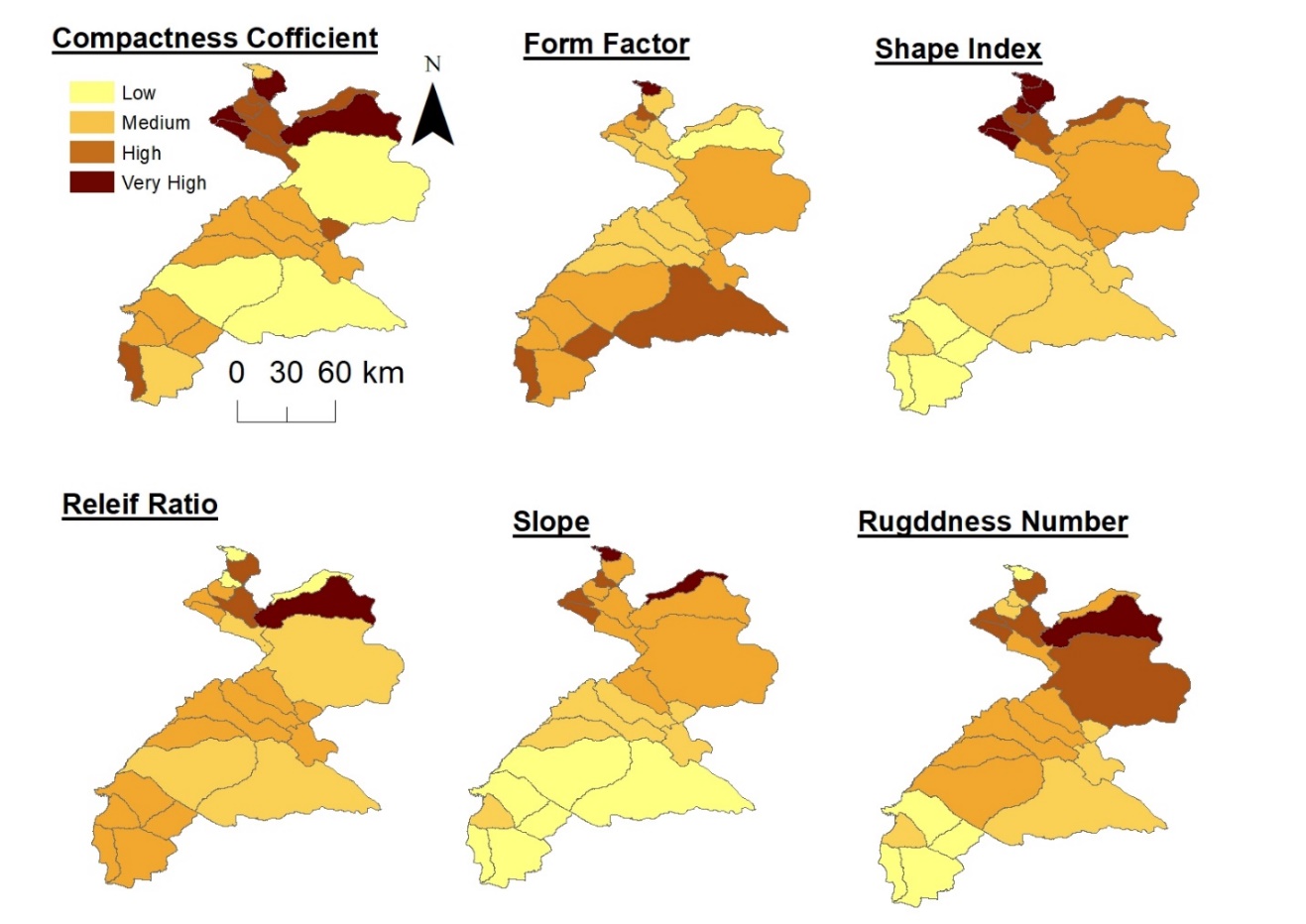


**Fig. S3** Average annual rainfall throughout the study area

|  |  |
| --- | --- |
|  |  |

**Fig. S4** Land use/cover change between 2018 and 2020


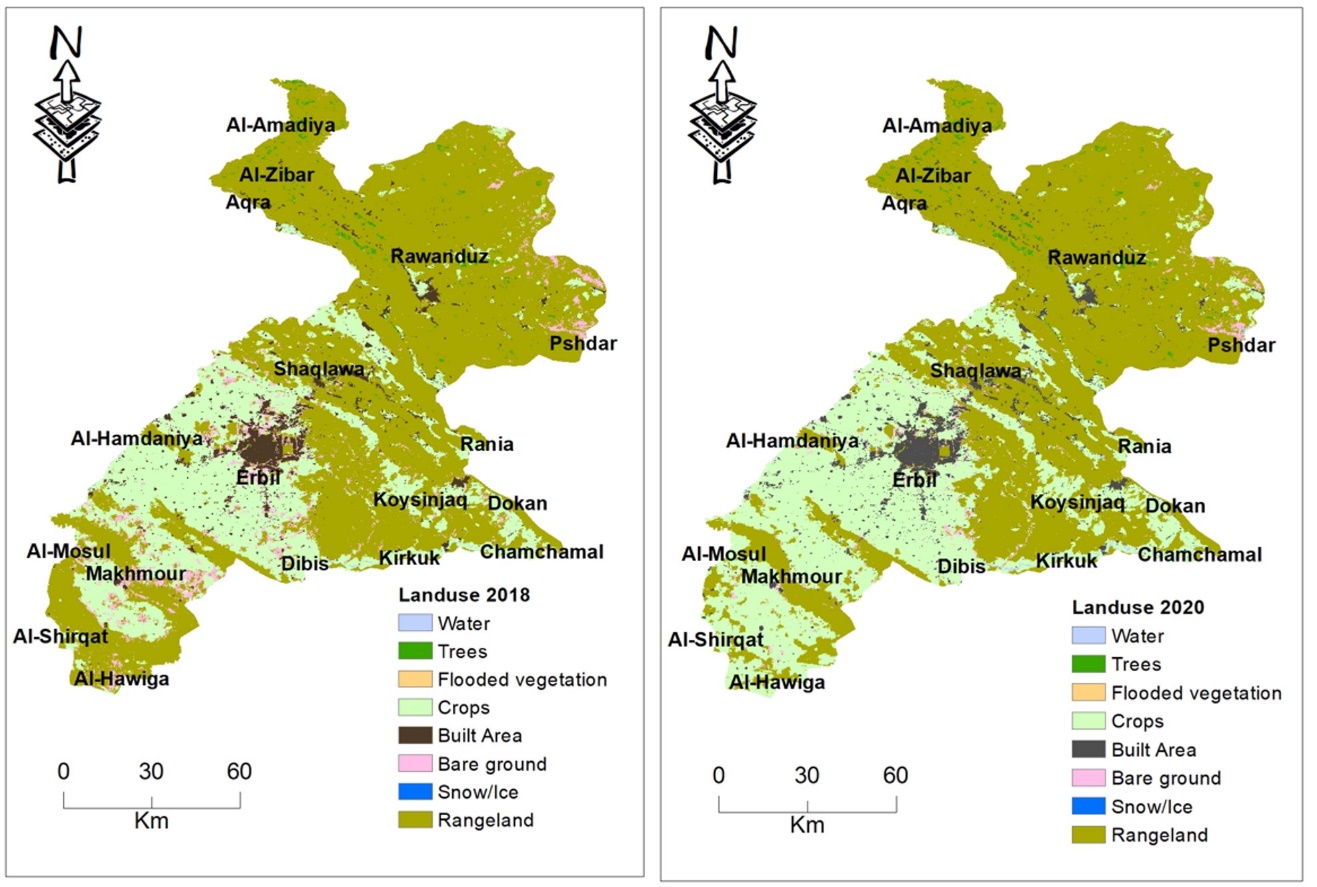

Supplement: Supplementary file 1 — Supplementary Information. [file 41598_2023_39290_MOESM1_ESM.docx]
